# Melatonin administration attenuates fibrosis progression in frozen shoulder syndrome: a rat model study

**DOI:** 10.1186/s12891-025-09198-3

**Published:** 2025-10-15

**Authors:** Serhat Gürbüz, Süleyman Semih Dedeoğlu, Ahmet Keskin, Mustafa Buğra Ayaz, Yunus Imren, Cem Comunoğlu, Bulent Karslıoğlu, Buse Aki

**Affiliations:** 1 Department of Orthopedics and Traumatology, Metin Sabancı Baltalimanı Bone Diseases Training and Research Center, Health Sciences University Turkey, İstanbul, Turkey; 2 Department of Orthopedics and Traumatology, Istinye University Turkey, Liv Hospital Vadistanbul, İstanbul, Turkey; 3 Depatment of Pathology, Health Sciences University Turkey, Prof Dr Cemil Tascioglu City Hospital, Istanbul, Turkey

**Keywords:** Frozen shoulder syndrome, Melatonin, Fibrosis, Animal model, Shoulder joint, Adhesive capsulitis, Contracture, Inflammation

## Abstract

**Background:**

Frozen shoulder syndrome, or adhesive capsulitis, is characterized by pain and restricted joint mobility due to fibrotic changes within the shoulder joint capsule. Despite its potential for spontaneous resolution, persistent symptoms often necessitate further treatment. Melatonin, known for its anti-inflammatory and anti-fibrotic properties, has emerged as a promising therapeutic agent for this condition. This study aimed to investigate the impact of melatonin on adhesive capsulitis in a rat model, focusing on its potential to attenuate fibrosis progression and improve joint pathology.

**Methods:**

Thirty female Sprague-Dawley rats were utilized in this study. Frozen shoulder syndrome was surgically induced, and the rats were divided into three groups: G1 receiving a melatonin antagonist, G2 receiving daily melatonin supplementation, and G3 without intervention. Histopathological evaluations were conducted at weeks 8 and 16 post-simulation to assess fibrosis, synovial hypertrophy, inflammatory cell infiltration, and hypervascularity. Statistical analyses were performed to determine significant differences between the groups.

**Results:**

Histological evaluations revealed that melatonin administration significantly attenuated fibrosis progression in the shoulder joint, particularly in the G2 (*p* < 0.05). No significant differences were observed in synovial hypertrophy, inflammatory cell infiltration, or hypervascularity between the groups (*p* > 0.05). Survival rates indicated no fatalities in the group receiving daily melatonin supplementation. Statistical analysis showed significant differences in fibrosis levels at 8 weeks between groups, with G1 exhibiting higher rate of severe fibrosis (*p* = 0.038). However, at 16 weeks, no significant differences were observed between groups (*p* > 0.05). Temporal changes within groups indicated a significant decrease in fibrosis levels and synovial hypertrophy from 8 to 16 weeks in G1 and G3 (*p* < 0.05). A comparison between operated and non-operated sides revealed significant differences in fibrosis levels in both groups at 8 and 16 weeks (*p* < 0.05).

**Conclusion:**

Melatonin administration demonstrated potential therapeutic benefits in attenuating fibrosis progression and reducing synovial hypertrophy in adhesive capsulitis.

**Level of evidence:**

NA, Animal Study

**Supplementary Information:**

The online version contains supplementary material available at 10.1186/s12891-025-09198-3.

## Introduction

Frozen shoulder syndrome, also known as adhesive capsulitis, is a challenging condition characterized by pain and restricted joint mobility due to fibrotic changes within the shoulder joint capsule [1]. Despite its potential for spontaneous resolution, the condition can persist, necessitating further treatment. Melatonin, recognized for its multifaceted effects including anti-inflammatory and anti-fibrotic properties, has emerged as a promising therapeutic agent for this syndrome [2, 3]. 

While debate persists regarding its etiology, frozen shoulder syndrome predominantly affects individuals over 40, with a higher prevalence among females and a notable incidence in the non-dominant shoulder [4, 5]. Although the syndrome often resolves spontaneously within 28 to 32 months, persistent joint function loss can occur.

Management strategies for frozen shoulder syndrome typically involve early interventions such as intra-articular corticosteroid injections and physical therapy modalities to alleviate symptoms and improve joint mobility [6, 7]. Melatonin’s anti-inflammatory effects have been demonstrated in both animal and human studies, showing a reduction in pro-inflammatory cytokines and an increase in anti-inflammatory cytokines [8]. However, melatonin has also found to have inflammatory effects at physiological doses. In our view melatonin can act as both an anti-inflammatory and a pro-inflammatory agent depending on the circumstances [9]. 

Moreover, melatonin’s anti-apoptotic properties and its ability to modulate fibroblast activity suggest its potential in mitigating fibrosis, as evidenced by clinical trials in conditions such as hypertrophic scars and hepatic fibrosis [2, 3, 8].

Considering the diverse therapeutic effects of melatonin, this study aims to investigate its impact on adhesive capsulitis in a rat model, focusing on its potential to attenuate fibrosis progression and enhance joint pathology. Understanding melatonin’s role in this context could provide novel insights into the treatment of frozen shoulder syndrome and pave the way for further clinical investigations in human subjects. Therefore, this study was designed to elucidate the therapeutic potential of melatonin in adhesive capsulitis, with the hypothesis that melatonin administration would mitigate fibrosis progression and improve joint pathology in the rat model.

## Materials and methods

### Study design

Ethical approval was obtained from the Acıbadem Mehmet Ali Aydinlar University animal research ethics committee, along with the necessary permissions. All experimental protocols were approved by Acıbadem Mehmet Ali Aydinlar University animal research institutional committee, all procedures were conducted in accordance with relevant guidelines and all methods were in accordance with ARRIVE guidelines.

**Fig. 1:**
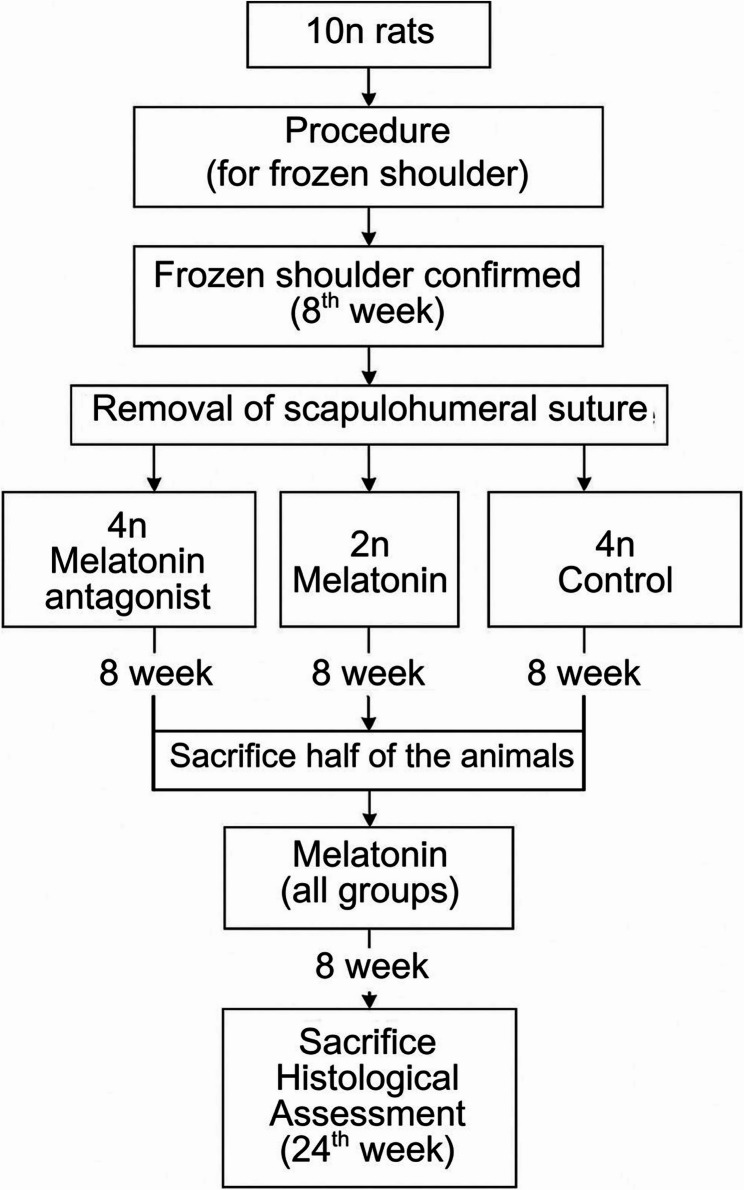
Study design diagram

The study was designed to include 3 groups of rats, all of which were modelled with frozen shoulder syndrome. This study was structured as a two-phase investigation. In the first phase, after the modelling of frozen shoulder syndrome, 1 st group received a melatonin antagonist, the 2nd group was administered melatonin, and the 3rd group was observed without any intervention as a control. (Figure 1) Following an 8-week follow-up period, Half of the subjects will be euthanized for histological evaluation. Subsequently, the second phase of the study will commence. During this phase, all remaining rats will be administered daily i.p. melatonin for a period of 8 weeks. At the end, a comprehensive evaluation of the experimental outcomes will be conducted.

Group 2 was designed to include 2n rats, whereas Groups 1 and 3 each included 4n rats. The smaller sample size in Group 2, which was to receive daily melatonin administration from beginning to end, was intentional, as all animals were planned to undergo daily intraperitoneal melatonin administration in the second phase of the study.

Disease simulation validation was conducted by monitoring both active and passive joint range of motion under sedation. The sample size was determined using G power analysis. Based on Okajima’s article as a reference for joint range of motion, the analysis indicated that a minimum of *n* = 3 rats per group was established for Power: 0.80 and α: 0.05 [10].

A total number of 30 Female Sprague-Dawley rats, weighing 230–300 g and aged 9 weeks, which were obtained from the Acıbadem Mehmet Ali Aydinlar University animal research laboratory were employed. Luzindole (Sigma-Aldrich, MO, USA), a melatonin receptor antagonist, was used in the first group [11].

From the eighth week onwards, after confirming shoulder joint movement restriction in all rats according to their initially assigned groups, they were all included in the study (Video 4) (Fig. 1).

Melatonin (Sigma-Aldrich), in powder form, was dissolved in 99% ethyl alcohol at a concentration of 10 mg/mL. This solution then served as a basis for subsequent procedures. Desired concentrations were calculated from this solution, which was then diluted with 0.9% saline before use. When Luzindole (Sigma-Aldrich) was needed, the substance was dissolved in a DMSO solution, and the resulting solution was directly administered to the rats. The dosages were determined based on literature references, with melatonin administered at 0.5 mg/kg/day and Luzindole at 0.2 mg/kg/day. After intraperitoneal injections, the injected rats were observed for 15 min each. Melatonin and Luzindole injections were administered 30 min before lights-off.

## Surgical technique

Surgical procedures were conducted using the technique described by Okajima et al. [10], with minor modifications rather than using other techniques described in literature [12–14]. Xylazine(0.15 cc) and ketamine(0.3 cc) were administered intraperitoneally for anesthesia. The surgical technique involved making an incision along the dorsum of the body, parallel to the humeral shaft, to access the scapula. (video1) Two holes were drilled into the scapula using a burr, through which a 2.0 Ethicon suture was passed and knotted by looping anteriorly from the humerus. (see videos 2,3) Unlike the original technique, in which sutures were passed through the lateral aspect of the scapula, in this study, sutures were passed through the medial aspect. (video 2) This modification aimed to benefit the thicker bone in the medial aspect to provide more resistance against potential fractures during burr usage and to ensure a safe distance from the brachial plexus and neurovascular bundle. Post-procedure, each rat received 0.5 ml of 0.9% isotonic solution intraperitoneally, followed by a three-day postoperative regimen of paracetamol(0.1 cc) and cefazolin(0.25 cc). After 8 weeks, through an additional surgical procedure, scapulo-humeral sutures were removed.

## Histopathological evaluation

The histopathological assessment aimed to explore the anti-fibrotic effects of melatonin on frozen shoulder syndrome [15]. Additionally, the effects of melatonin on vascular remodeling, synovial hypertrophy, and inflammatory cell infiltration were evaluated [16]. 

All euthanasias were performed by administering high doses of anesthetic agents. Bilateral shoulder girdles in all euthanized rats, including the scapulae and clavicle bones, were amputated and examined.

All histopathological examinations were conducted at the pathology clinic laboratory of the hospital. Samples were macroscopically evaluated and fixed in a 10% formalin solution. Subsequently, specimens were decalcified and then embedded in paraffin tissue blocks. For histopathological evaluation 2–3 μm thick sections were cut from formalin fixed paraffin embedded (FFPE) tissue blocks. All sections stained with hematoxylin eosin and Masson trichrome (Biooptica). The examination of fibrosis was conducted by Masson trichrome [17]. Two independent examiners conducted the assessments to minimize interobserver bias.

All specimens were examined using an Olympus BX5-FL microscope under x200 magnification. For each subject the whole surface area of the extracted specimen was assesed. All specimens were graded on a scale from 0 to 3, where 0 indicated no inflammatory cells, no fibrosis, and no increase in vascularity, while 3 represented severe fibrosis, abundant inflammatory cells, and a dramatic increase in angiogenesis. In the evaluation of synovial hypertrophy score 0 was assigned to 0–1 lined cells, score 1 to 2–4 lined cells, score 2 to 5–9 lined cells and score 3 to over 10 lines of synovial cells.

Histopathological examination was conducted on the euthanasied rats in such a manner that it would be compared with both the experimentally induced fibrosis side and the healthy side. At week 24, all remaining rats were euthanasied for a comprehensive histopathological examination.

**Fig. 2:**
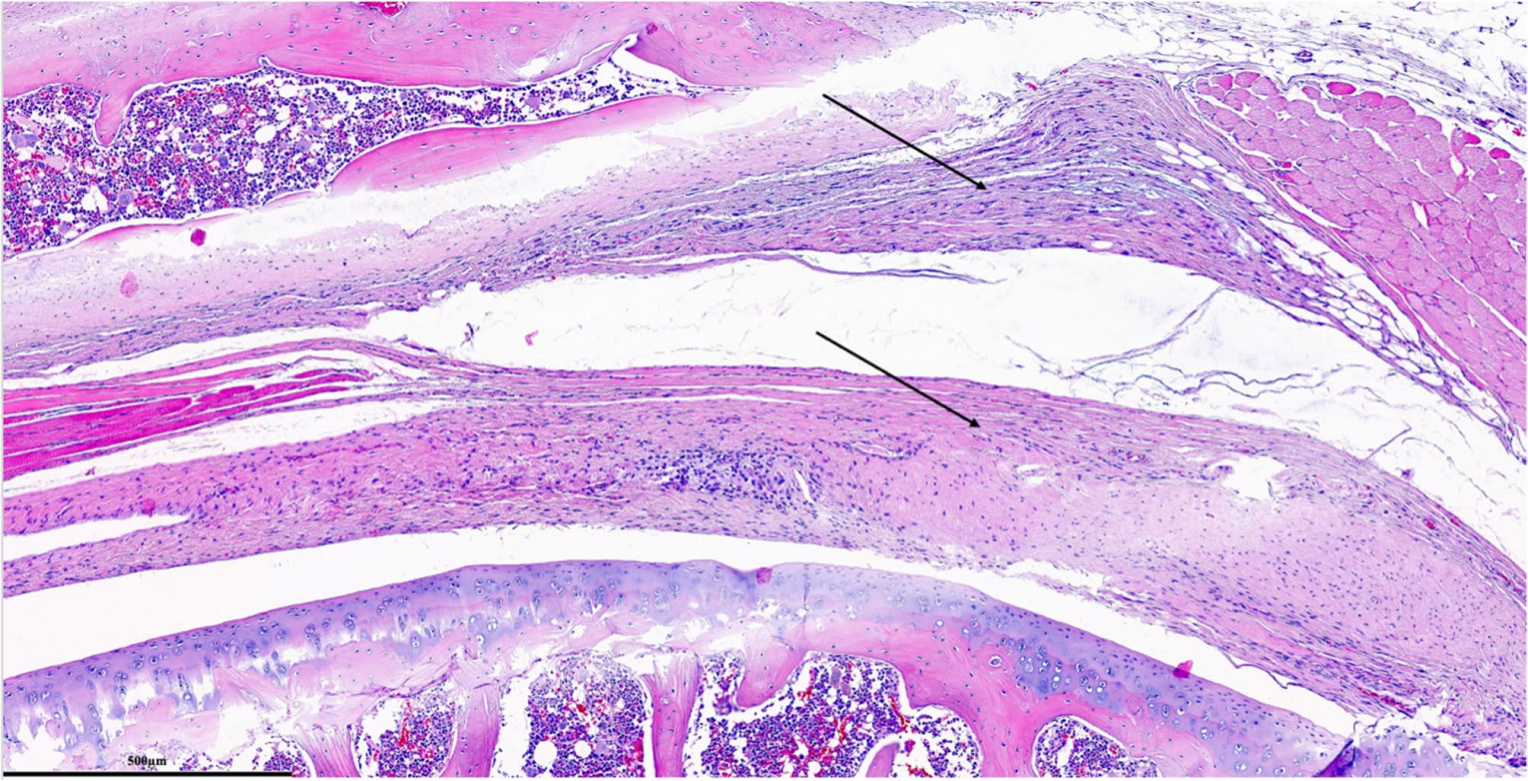
Specimen from Group 1, 16^th^ week (H&E x100), Prominent areas of fibrosis are indicated by arrows

**Fig. 3:**
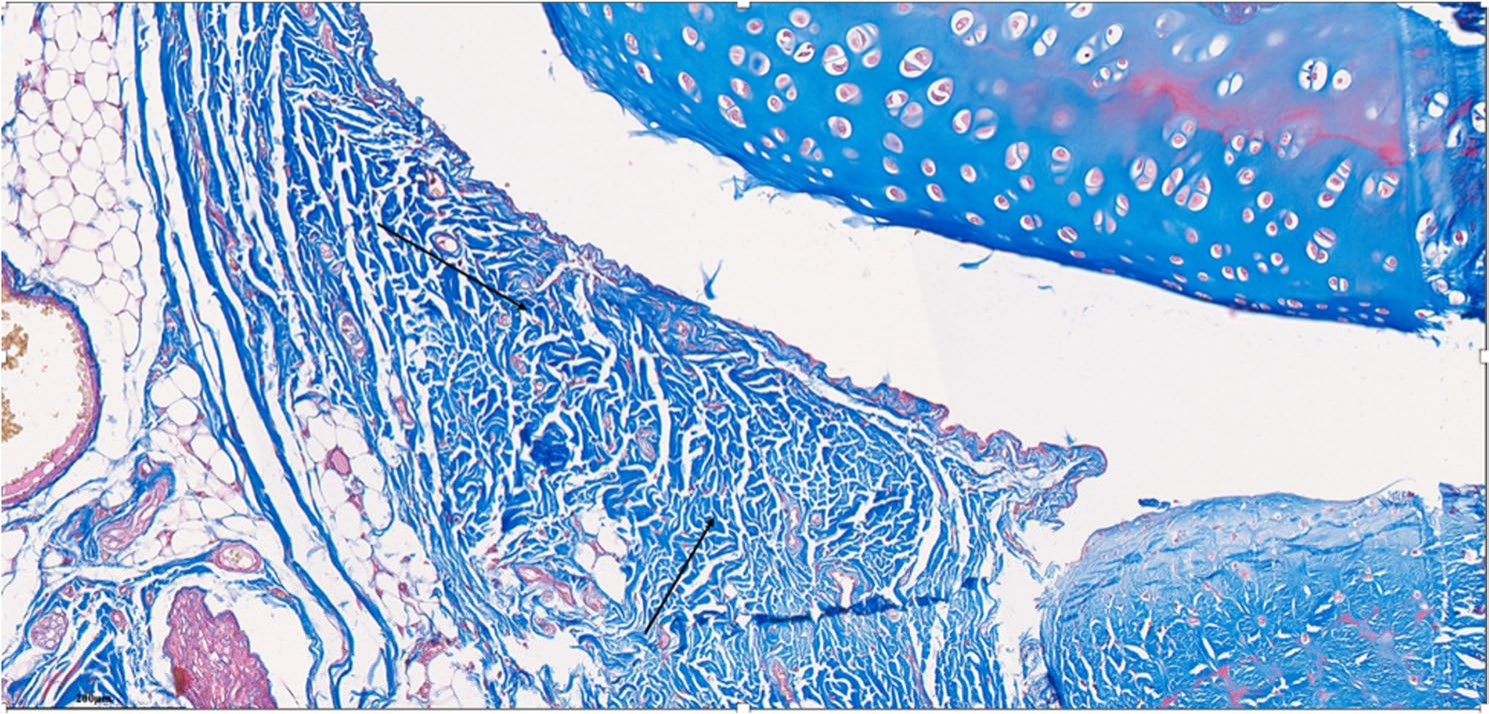
Specimen from Group 1, 16^th^ week (MTx200), Prominent areas of fibrosis are indicated by arrows

**Fig. 4:**
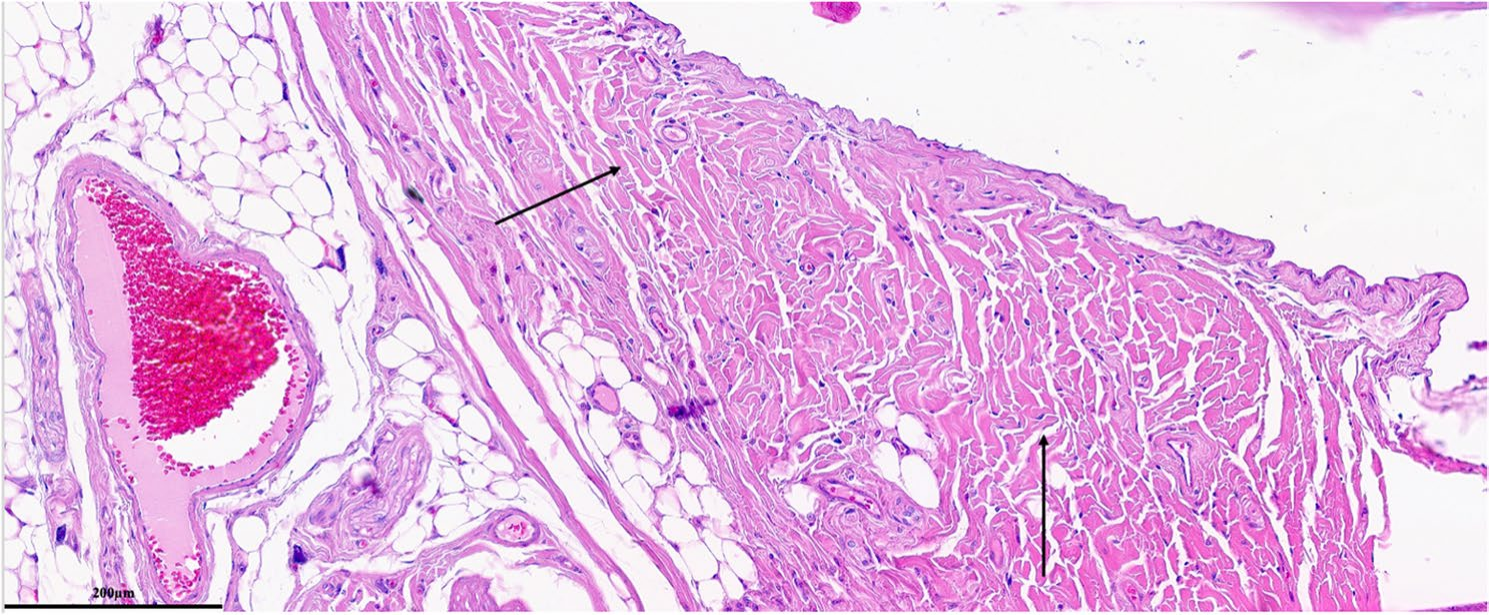
Specimen from Group 1, 24^th^ week (H&E x200), Prominent areas of fibrosis are indicated by arrows

**Fig. 5:**
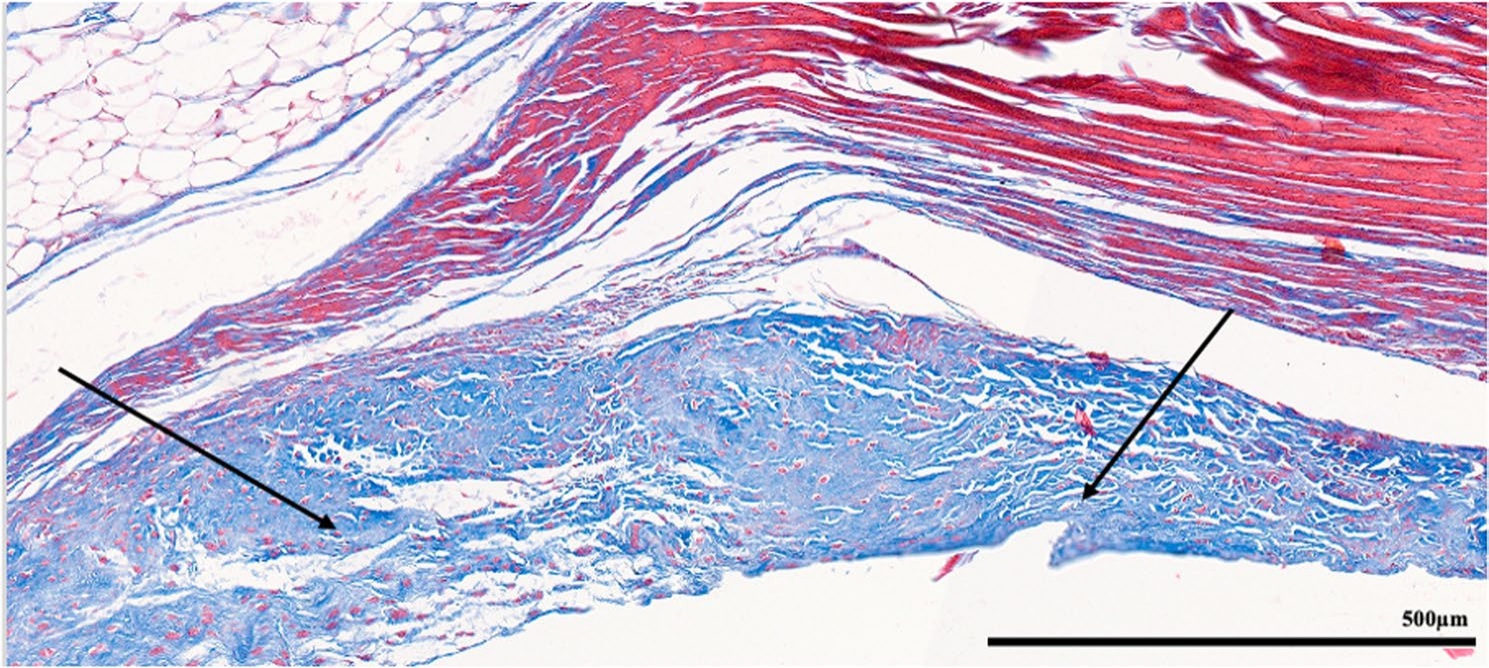
Specimen from Group 1, 24^th^ week (MTx200), Prominent areas of fibrosis are indicated by arrows

**Fig. 6:**
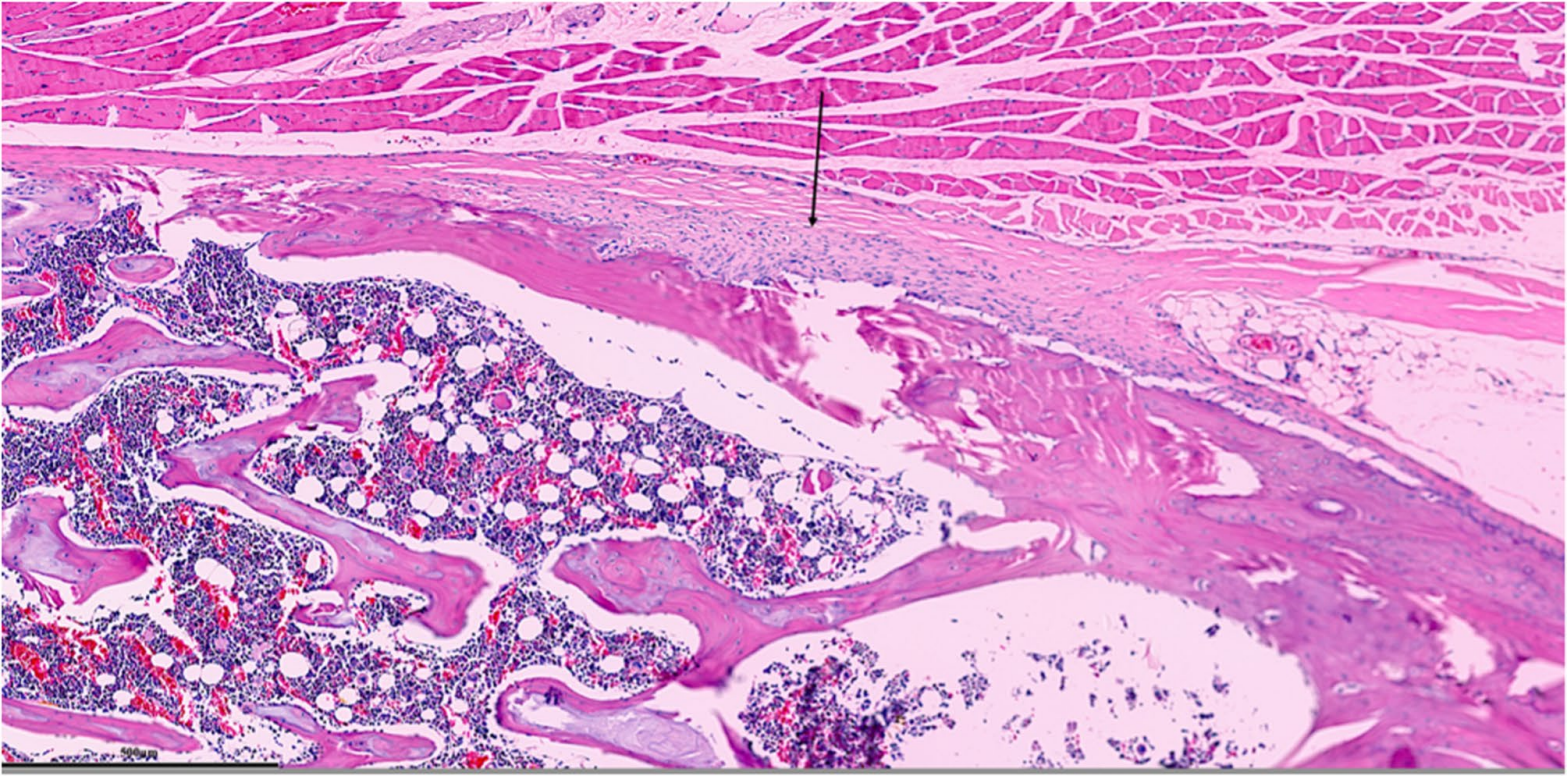
Specimen from Group 2, 16^th^ week (H&E x100), Prominent fibrotic area is indicated by an arrow

**Fig. 7:**
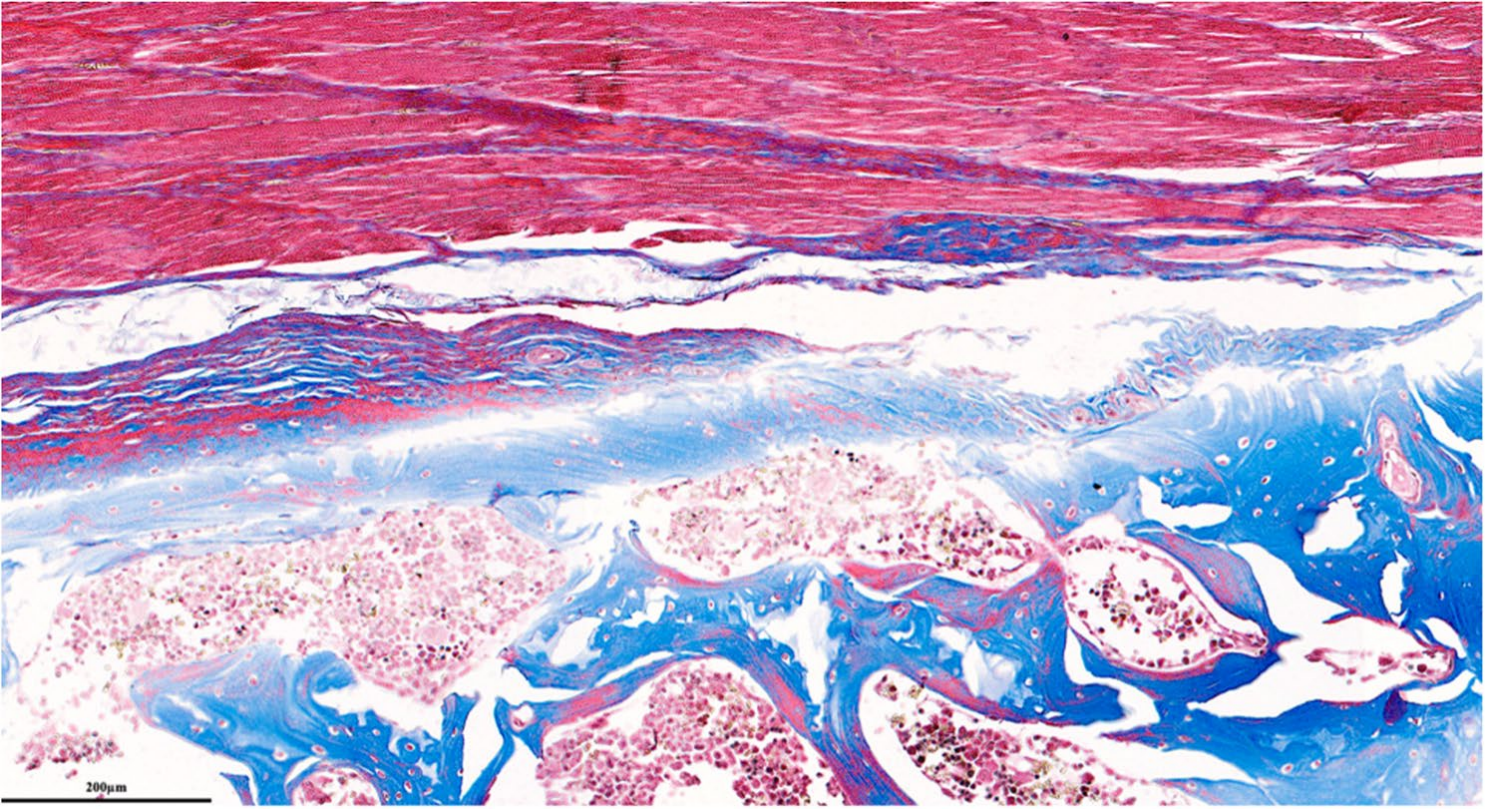
Specimen from Group 2, 16th week (MT x100)

**Fig. 8:**
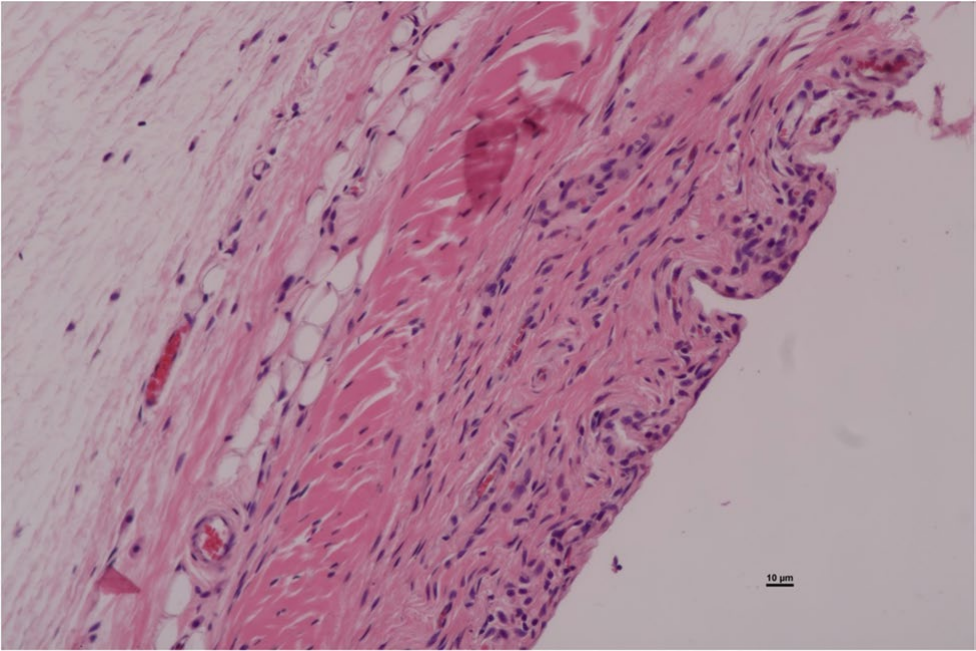
Specimen from Group 2, 24th week (H&E x100)

**Fig. 9:**
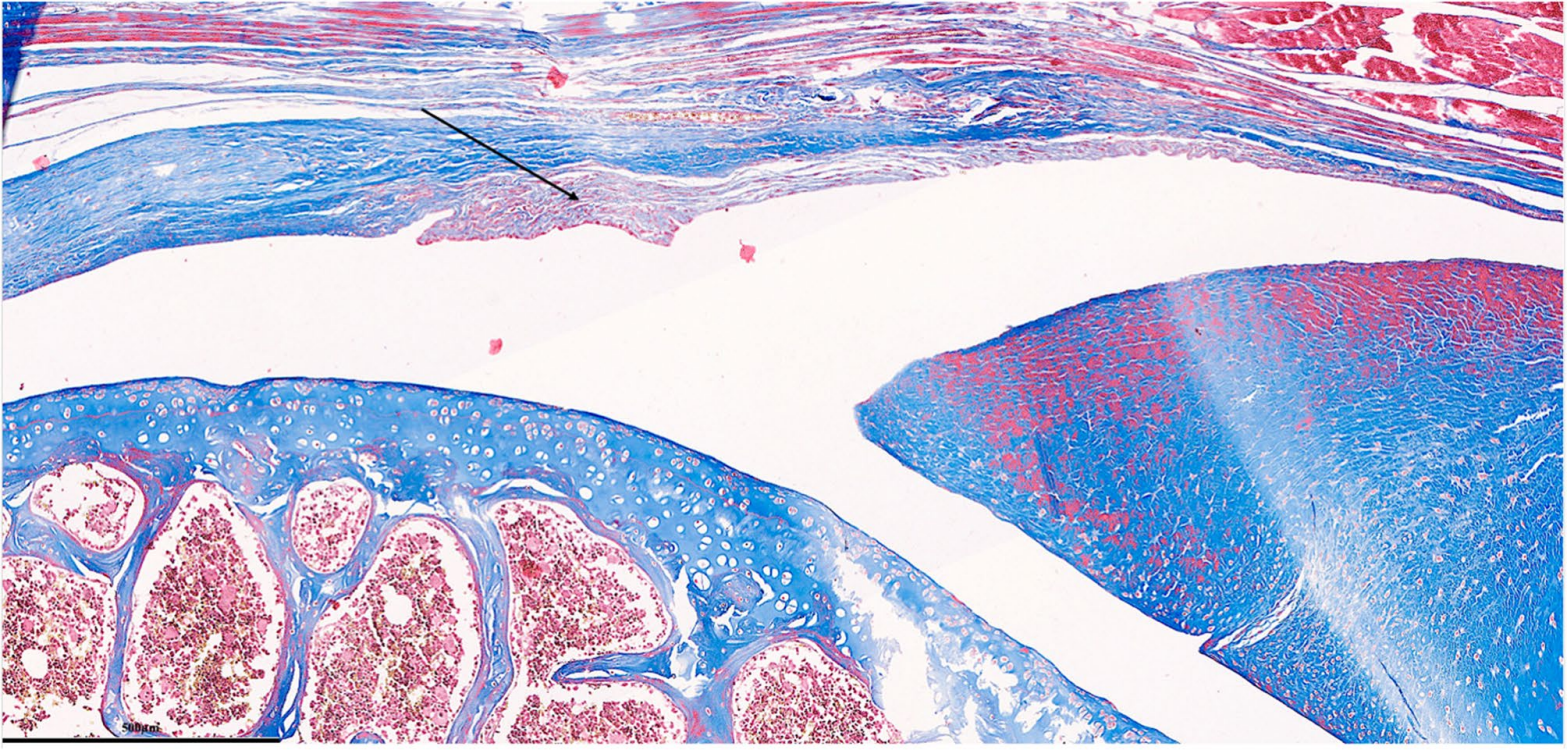
Specimen from Group 2, 24th week (MT x200), A region of prominent synovial hyperplasia is shown by an arrow

**Fig. 10:**
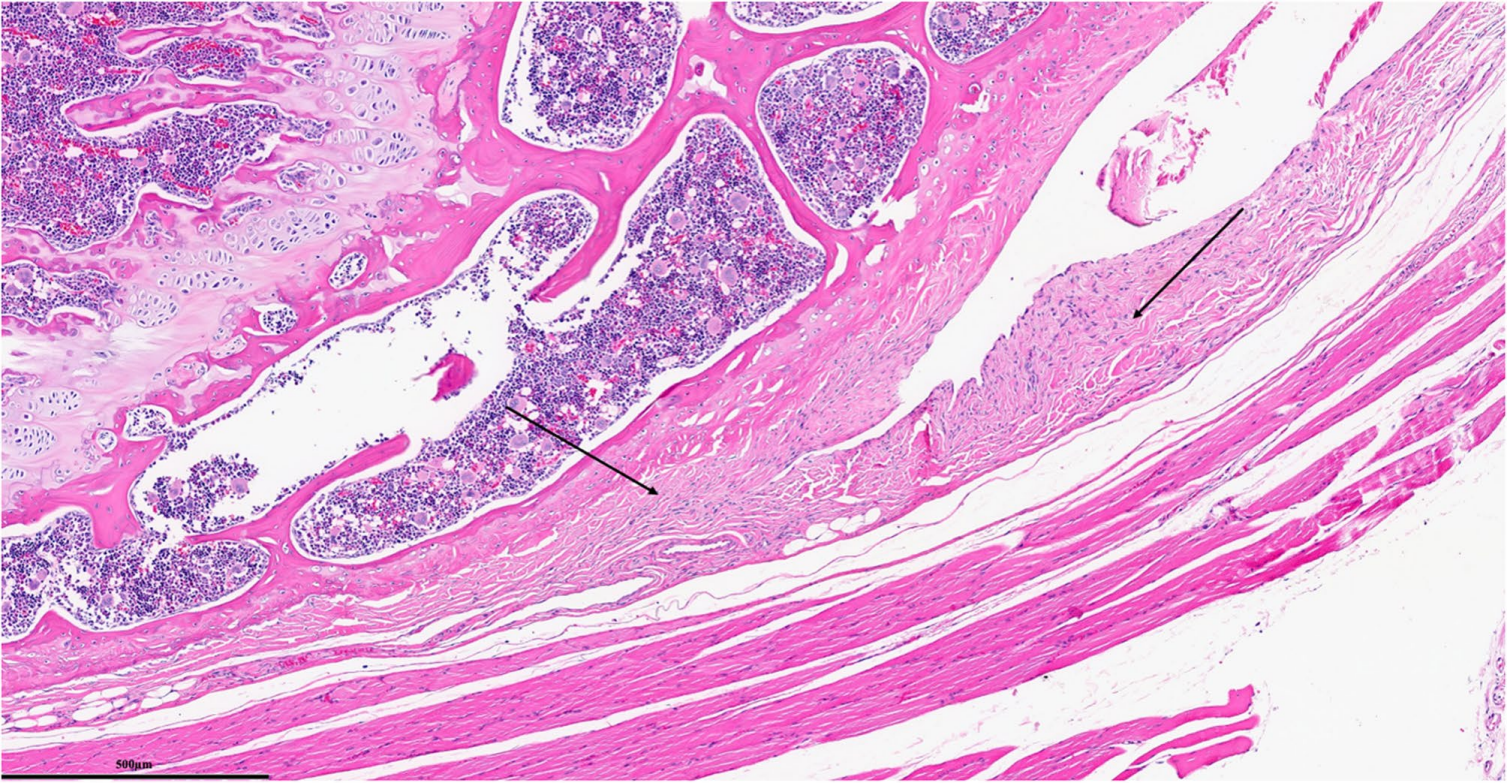
Specimen from Group 3, 16^th^ week (H&E x100), Prominent areas of fibrosis are indicated by arrows

**Fig. 11:**
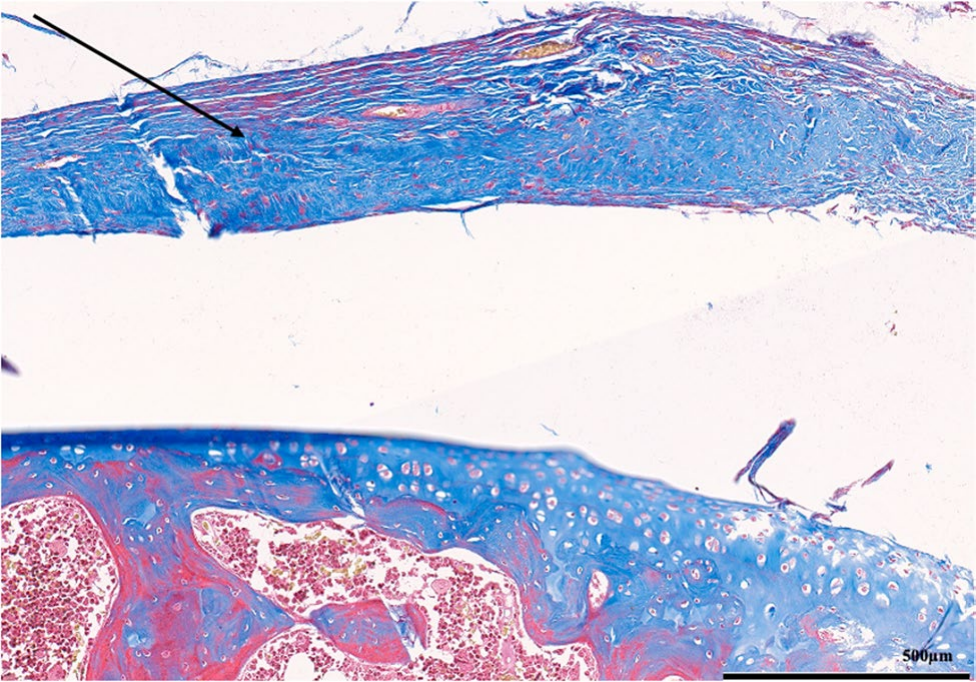
Specimen from Group 3, 16^th^ week (MT x200), Prominent fibrotic area is indicated by an arrow

**Fig. 12:**
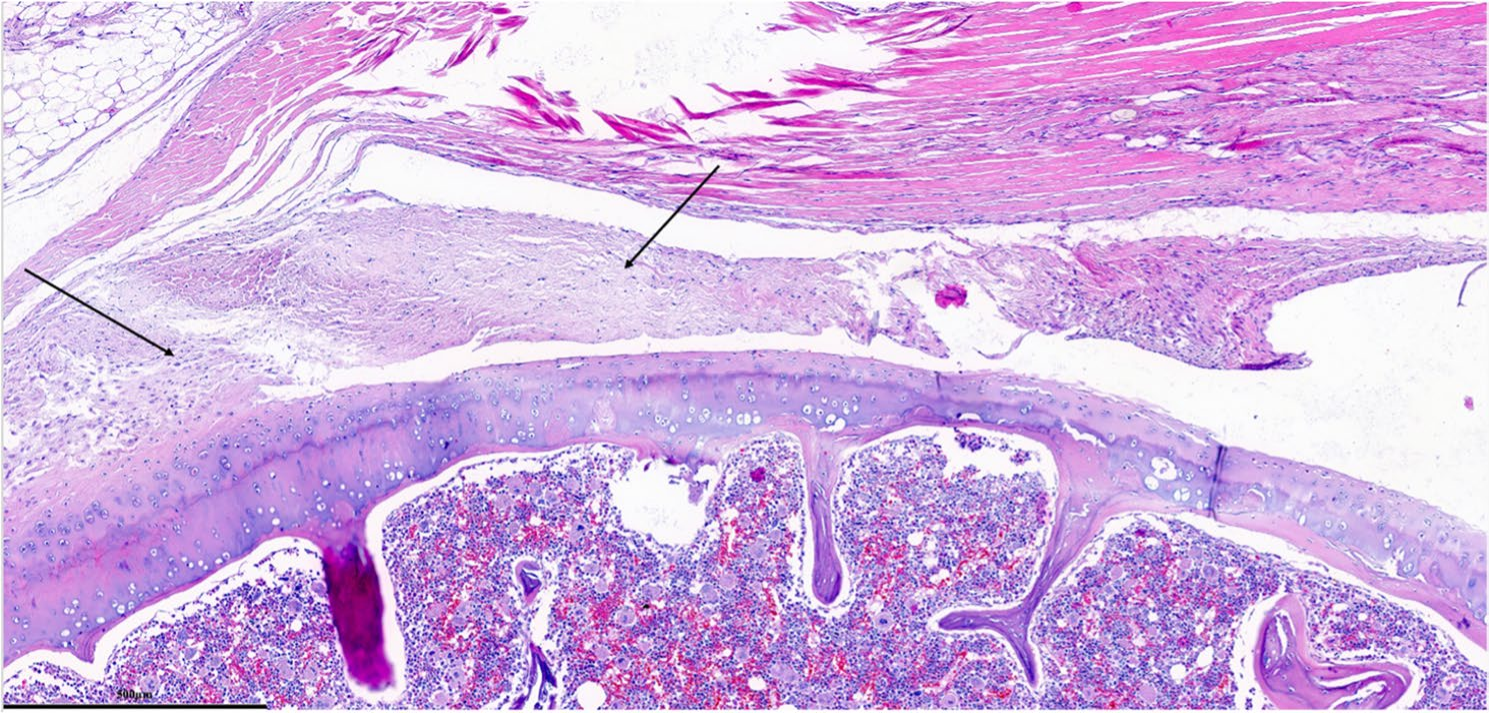
Specimen from Group 3, 24^th^ week (H&E x100), Areas of synovial fibrosis are shown by arrows

**Fig. 13:**
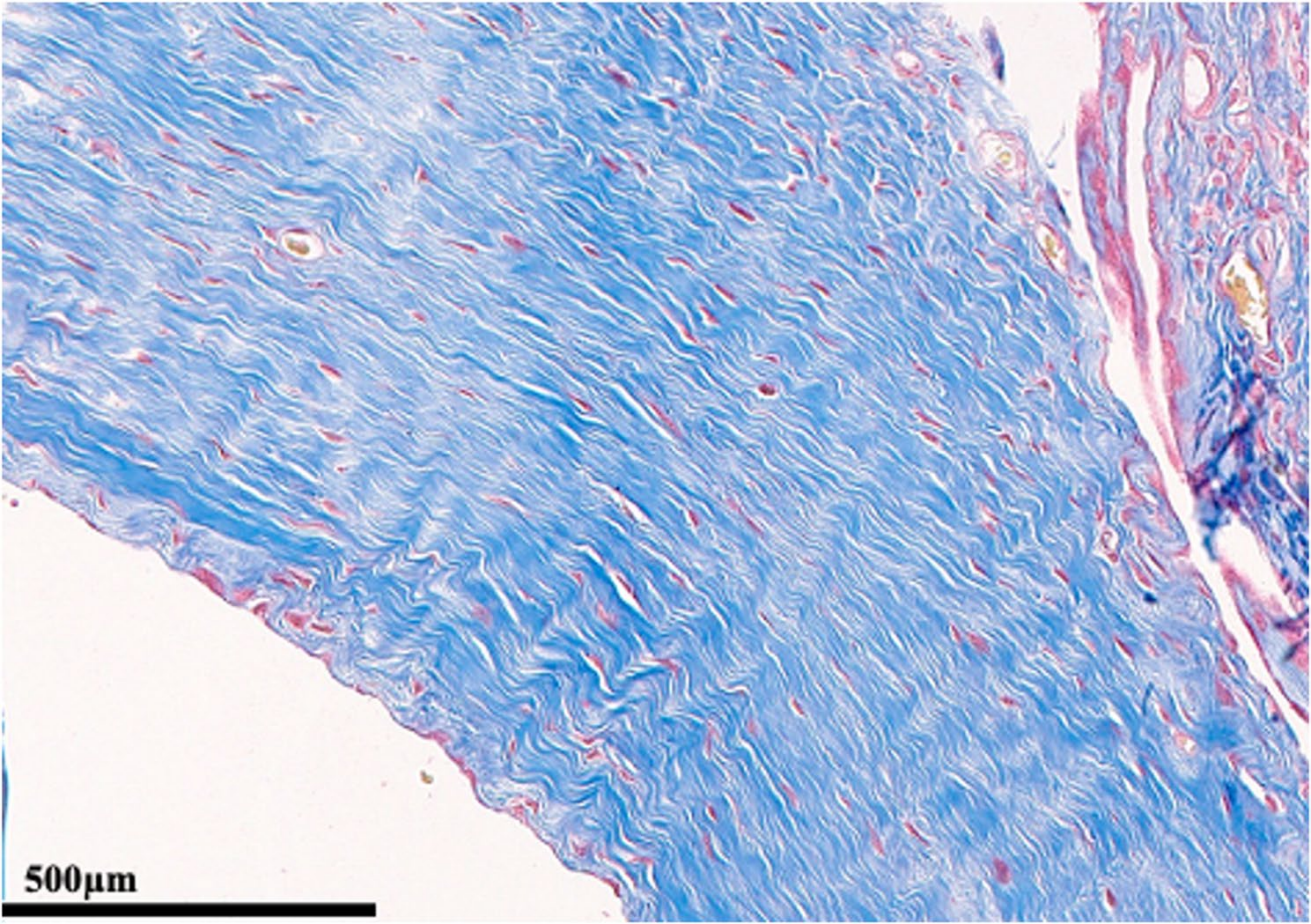
Specimen from Group 3, 24th week (MT x200)

Each parameter was graded on a scale of 0 to 3 according to its severity (0: None; 1: Mild; 2: Moderate; 3: Severe), and a total score was assigned based on this grading system. (Figures 2, 3, 4, 5, 6, 7, 8, 9, 10, 11, 12 and 13) During the euthanasies conducted at the sixteenth week in all three groups, preparations were evaluated for fibrosis, synovial hypertrophy, inflammatory cell infiltration, and hypervascularity. Each parameter was scored according to severity, ranging from 0 to 3, and a total score was calculated for each preparation.

### Statistical analysis

Statistical analysis was performed using IBM SPSS Statistics 22 software, employing non-parametric tests due to the non-normal distribution of parameters. The conformity of the parameters to normal distribution was evaluated using the Shapiro-Wilk test, and it was determined that the parameters did not follow a normal distribution. In the evaluation of study data, descriptive statistical methods (mean, standard deviation, median, minimum, maximum, frequency) were used. For the comparison of quantitative data between groups, the Kruskal-Wallis test was applied, and Dunn’s test was used to identify the group responsible for the observed difference. For comparisons of parameters at the 16th and 24th weeks, the Mann-Whitney U test was employed. The Wilcoxon signed-rank test was used for comparisons between the affected and unaffected sides. For categorical data comparisons, the Chi-square test, Fisher’s Exact Chi-square test, and the Fisher-Freeman-Halton Exact Chi-square test were utilized. Statistical significance was set at *p* < 0.05.

## Results

The study, which initially commenced with 30 rats, concluded with a total of 27 rats due to three losses during the experimentation period. Two rats subjected to the melatonin receptor antagonist and one rat from the control group did not survive. No fatalities occurred in the group recieving daily intraperitoneal administration of melatonin. All fatalities occurred in between 8th and 16th week. During the initial histopathological examination, an equal number of rats from Groups 1 (*n* = 5) and 3 (*n* = 5), and 3 rats from Group 2 were euthanized. At week 24, all remaining rats, including 5 rats from Group 1, 3 rats from Group 2, and 6 rats from Group 3, were euthanized.

Additionally, an ovarian mass was detected in one rat from the first group in the seventh post-procedure week; however, since it did not affect shoulder joint mobility, the rat was included in the study.

There was a statistically significant difference between the groups (*p* = 0.038) in fibrosis levels at sixteen weeks.(Table 1) Group 1 exhibited significantly higher rates of severe fibrosis compared to Groups 2 and 3. Group 2 showed significantly higher rates of mild fibrosis compared to Groups 1 and 3. Group 3 Had significantly higher rates of moderate fibrosis compared to Groups 1 and 2. No significant differences were observed between the groups in fibrosis levels at 24 weeks (Table 2).

**Table 1:** Evaluation of the groups (intervened side) in terms of 16th week outcomes

16th Week		Group 1 (*n* = 5)	Group 2 (*n* = 3)	Group 3 (*n* = 5)	
		*n* (%)	*n* (%)	*n* (%)	*p*
Fibrosis	None	0 (%0)	1 (%33,3)	0 (%0)	0,038*
	Mild	0 (%0)	2 (%66,7)	1 (%20)	
	Moderate	1 (%20)	0 (%0)	3 (%60)	
	Severe	4 (%80)	0 (%0)	1 (%20)	
Synovial Hypertrophy	None	5 (%100)	3 (%100)	2 (%40)	0,248
	Mild	0 (%0)	0 (%0)	1 (%20)	
	Moderate	0 (%0)	0 (%0)	2 (%40)	
Inflammatory Cell Infiltration	None	1 (%20)	3 (%100)	3 (%60)	0,209
	Mild	2 (%40)	0 (%0)	1 (%20)	
	Moderate	0 (%0)	0 (%0)	1 (%20)	
	Severe	2 (%40)	0 (%0)	0 (%0)	
Hypervascularity	None	4 (%80)	0 (%0)	4 (%80)	0,193
	Mild	0 (%0)	1 (%33,3)	0 (%0)	
	Moderate	0 (%0)	1 (%33,3)	1 (%20)	
	Severe	1 (%20)	1 (%33,3)	0 (%0)	

*p < 0.05

**Table 2:** Histopathological evaluation of intervened shoulder

		16th week	24th week	
		M ± SD (median)	M ± SD (median)	*p*
Intervened Side		(Min-Max)	(Min-Max)	
Fibrosis	Group 1	3 ± 0 (3)	1 ± 1 (1)	0.014*
		(3–3)	(0–2)	
	Group 2	1.7 ± 1.5 (2)	1 ± 1 (1)	0.637
		(0–3)	(0–2)	
	Group 3	2.2 ± 0,4 (2)	0.3 ± 0.5 (0)	0.008*
		(2–3)	(0–1)	
Synovial Hypertrophy	Group 1	0 ± 0 (0)	0 ± 0 (0)	1.000
		(0–0)	(0–0)	
	Group 2	0 ± 0 (0)	0 ± 0 (0)	1.000
		(0–0)	(0–0)	
	Group 3	0.8 ± 1,1 (0)	0 ± 0 (0)	0.036*
		(0–2)	(0–0)	
Inflammatory Cell Infiltration	Group 1	1.2 ± 1.6 (0)	1 ± 1.7 (0)	0.588
		(0–3)	(0–3)	
	Group 2	0 ± 0 (0)	0.7 ± 1.2 (0)	0.317
		(0–0)	(0–2)	
	Group 3	0.2 ± 0.4 (0)	0.2 ± 0.4 (0)	0.351
		(0–1)	(0–1)	
Hypervascularity	Group 1	0.6 ± 1.3 (0)	0 ± 0 (0)	0.439
		(0–3)	(0–0)	
	Group 2	1.3 ± 1.2 (2)	0.7 ± 1.2 (0)	0.456
		(0–2)	(0–2)	
	Group 3	0.4 ± 0.9 (0)	0.7 ± 0.8 (0.5)	0.456
		(0–2)	(0–1)	
Total score	Group 1	4.8 ± 2,7 (3)	2 ± 1 (2)	0.084
		(3–9)	(1–3)	
	Group 2	3 ± 1.7 (2)	2.3 ± 2.1 (3)	0.825
		(2–5)	(0–4)	
	Group 3	3.6 ± 1.7 (4)	1.2 ± 0.8 (1)	0.009*
		(2–6)	(0–2)	

*p < 0.05

In the second group of animals (*n* = 6), which received daily intraperitoneal melatonin, no losses were observed. In the third group (*n* = 11), which Had ad libitum access to water and food, the study continued with a total of 11 rats despite the death of one rat. In the first group.

(*n* = 10), one animal developed necrosis, suspected to be ischemia-related, on the operated side. Generally, during wound follow-ups and at injection sites, infectious complications were more frequently observed in the first group (four cases total, one in the control group and none in the melatonin-treated group), which received Luzindole, for the duration of their treatment. Fortunately, these complications were managed with medications and regular dressings.

No statistically significant differences were observed between the groups regarding synovial hypertrophy levels at 16 and 24 weeks. Additionally, no statistically significant differences were observed between the groups for inflammatory cell infiltration levels at 24 weeks. Similarly, hypervascularity levels at 24 weeks did not differ significantly between the groups. Finally, the total scores at 16 and 24 weeks were not statistically significant.(Table 3).

**Table 3:** Histopathological evaluation of the intervened side with regard to groups

		Group 1	Group 2	Group 3	
		M ± SD (median)	M ± SD (median)	M ± SD (median)	*p*
Intervened Side		(Min-Max)	(Min-Max)	(Min-Max)	
Fibrosis	16.week	2.8 ± 0.45 (3)	0.7 ± 0.58 (2)	2.0 ± 0.71 (2)	0.016*
		(2–3)	(0–1)	(1–3)	
	24.week	1 ± 1 (1)	1 ± 1 (1)	0.3 ± 0.5 (0)	0.130
		(0–2)	(0–2)	(0–1)	
Synovial Hypertrophy	16.week	0 ± 0 (0)	0 ± 0 (0)	0.8 ± 1.1 (0)	0.059
		(0–0)	(0–0)	(0–2)	
	24.week	0 ± 0 (0)	0 ± 0 (0)	0 ± 0 (0)	1.000
		(0–0)	(0–0)	(0–0)	
Inflammatory Cell Infiltration	16.week	1.2 ± 1.6 (0)	0 ± 0 (0)	0.2 ± 0.4 (0)	0.102
		(0–3)	(0–0)	(0–1)	
	24.week	1 ± 1.7 (0)	0.7 ± 1.2 (0)	0.2 ± 0.4 (0)	0.275
		(0–3)	(0–2)	(0–1)	
Hypervascularity	16.week	0.6 ± 1.3 (0)	1.3 ± 1.2 (2)	0.4 ± 0.9 (0)	0.102
		(0–3)	(0–2)	(0–2)	
	24.week	0 ± 0 (0)	0.7 ± 1.2 (0)	0.7 ± 0.8 (0.5)	0.236
		(0–0)	(0–2)	(0–2)	
Total score	16.week	4.8 ± 2.7 (3)	3 ± 1.7 (2)	3.6 ± 1.7 (4)	0.278
		(3–9)	(2–4)	(2–7)	
	24.week	2 ± 1 (2)	2.3 ± 2.1 (3)	1.2 ± 0.8 (1)	0.173
		(1–4)	(0–4)	(0–2)	

*p < 0.05

In Group 1, fibrosis levels decreased significantly from 16 to 24 weeks (*p* = 0.014). In Group 3, both fibrosis and synovial hypertrophy levels decreased significantly from 16 to 24 weeks (*p* = 0.008 and *p* = 0.036, respectively)(Table 2). Total scores also decreased significantly from 16 to 24 weeks (*p* = 0.009). In Group 1, fibrosis levels were significantly higher on the operated side at 16 weeks compared to the non-operated side (*p* = 0.034), moreover, total scores were also significantly higher on the operated side at 16 weeks (*p* = 0.041). (Table 4) On the other Hand, the comparison between shoulders did not show significant difference in group 2. (Table 5) In Group 3, both fibrosis levels and total scores were significantly higher on the operated side at both 16 and 24 weeks (*p* = 0.039 and *p* = 0.042 at 16 weeks; *p* = 0.034 at 24 weeks) (Tables 4 and 6). Similarly, in the third group, which served as the control group, statistically significant differences were found in fibrosis levels in the histopathological evaluations following the first and second euthanasies. Additionally, the level of synovial hypertrophy in this group at week 24 was statistically significantly lower than at week 16, indicating the therapeutic effect of melatonin on synovial hypertrophy. The total score between the 16th and 24th weeks in the histopathological evaluations also showed a statistically significant decrease in this group, suggesting the overall positive effect of melatonin on the condition.

**Table 4:** Comparison between intervened shoulder and control side in group 1

		Intervened Side	Control Side	
		Av.±SD (median)	Av.±SD (median)	*p*
Group 1		(Min-Max)	(Min-Max)	
Fibrosis	16.week	2,8 ± 0,45 (3)	0,6 ± 0,5 (1)	0,034*
		(2–3)	(0–1)	
	24.week	1,4 ± 0,9 (2)	0 ± 0 (0)	0,059
		(0–2)	(0–0)	
Synovial Hypertrophy	16.week	0 ± 0 (0)	0 ± 0 (0)	1,000
		(0–0)	(0–0)	
	24.week	0 ± 0 (0)	0 ± 0 (0)	1,000
		(0–0)	(0–0)	
Inflammatory Cell Infiltration	16.week	1,6 ± 1,3 (1)	0,2 ± 0,4 (0)	0,066
		(0–3)	(0–1)	
	24.week	1,2 ± 1,3 (1)	0 ± 0 (0)	0,109
		(0–3)	(0–0)	
Hypervascularity	16.week	0,6 ± 1,3 (0)	0,2 ± 0,4 (0)	0,317
		(0–3)	(0–1)	
	24.week	0 ± 0 (0)	0,4 ± 0,9 (0)	0,317
		(0–0)	(0–2)	
Total Score	16.week	5,0 ± 2,5 (4)	1 ± 1 (1)	0,041*
		(3–9)	(0–2)	
	24.week	2,6 ± 1,1 (3)	0,4 ± 0,9 (0)	0,066
		(1–4)	(0–2)	

*p < 0.05

**Table 5:** Comparison between intervened shoulder and control side in group 2

		Intervened Side	Control Side	
		Av.±SD (median)	Av.±SD (median)	*p*
Group 2		(Min-Max)	(Min-Max)	
Fibrosis	16.week	0,7 ± 0,58 (1)	0 ± 0 (0)	0,157
		(0–1)	(0–0)	
	24.week	1 ± 1 (1)	0,7 ± 0,6 (1)	0,317
		(0–2)	(0–1)	
Synovial Hypertrophy	16.week	0 ± 0 (0)	0 ± 0 (0)	1,000
		(0–0)	(0–0)	
	24. week	0 ± 0 (0)	0 ± 0 (0)	1,000
		(0–0)	(0–0)	
Inflammatory Cell Infiltration	16. week	0 ± 0 (0)	0 ± 0 (0)	1,000
		(0–0)	(0–0)	
	24. week	0,7 ± 1,2 (0)	0 ± 0 (0)	0,317
		(0–2)	(0–0)	
Hypervascularity	16. week	2,0 ± 1 (2)	0 ± 0 (0)	0,109
		(1–3)	(0–0)	
	24. week	0,7 ± 1,2 (0)	0 ± 0 (0)	0,317
		(0–2)	(0–0)	
Total Score	16. week	2,7 ± 1,2 (2)	0 ± 0 (0)	0,102
		(2–4)	(0–0)	
	24. week	2,3 ± 2,1 (3)	0,7 ± 0,6 (1)	0,180
		(0–4)	(0–1)

**Table 6:** Comparison between intervened side and control side in group 3

		Intervened Side	Control Side	
		Av.±SD (median)	Av.±SD (median)	*p*
Group 3		(Min-Max)	(Min-Max)	
Fibrosis	16.week	2,0 ± 0,71 (2)	0 ± 0 (0)	0,039*
		(1–3)	(0–0)	
	24.week	0,3 ± 0,5 (0)	0,2 ± 0,4 (0)	0,564
		(0–1)	(0–1)	
Synovial Hypertrophy	16.week	1,0 ± 1,0 (1)	0 ± 0 (0)	0,102
		(0–2)	(0–0)	
	24.week	0 ± 0 (0)	0 ± 0 (0)	1,000
		(0–0)	(0–0)	
Inflammatory Cell Infiltration	16.week	0,6 ± 0,9 (0)	0,2 ± 0,4 (0)	0,414
		(0–2)	(0–1)	
	24.week	0,2 ± 0,4 (0)	0 ± 0 (0)	0,317
		(0–1)	(0–0)	
Hypervascularity	16.week	0,4 ± 0,9 (0)	0,2 ± 0,4 (0)	0,655
		(0–2)	(0–1)	
	24.week	0,7 ± 0,8 (0,5)	0 ± 0 (0)	0,102
		(0–2)	(0–0)	
Total Score	16.week	4,2 ± 1,9 (4)	0,4 ± 0,5 (0)	0,042*
		(2–7)	(0–1)	
	24.week	1,2 ± 0,8 (1)	0,2 ± 0,4 (0)	0,034*
		(0–2)	(0–1)	

*p < 0.05

## Discussion

Adhesive capsulitis is primarily characterized by progressive fibrosis in the shoulder joint capsule. Given melatonin’s promising role in mitigating fibrotic processes in various conditions, exploring its impact on adhesive capsulitis could provide novel insights into its treatment [18–20]. In this study, melatonin administration significantly attenuated fibrosis in the shoulder joint. These findings highlight melatonin’s potential therapeutic role in adhesive capsulitis.

Although melatonin is widely recognized for its role in circadian rhythm regulation and sleep promotion, its biological actions extend far beyond these domains [21, 22]. Indeed, evidence suggests that melatonin’s relationship with sleep disorders in humans may be correlational rather than causal. For example, preserved circadian rhythms have been documented in individuals with pinealectomy or tetraplegia, and in patients taking non-selective beta blockers [21, 23]. These observations support the hypothesis that melatonin functions as a pleiotropic molecule with physiological effects that transcend its role in sleep regulation. This study, however, focuses on the orthopedic implications of melatonin in frozen shoulder syndrome, a condition primarily characterized by fibrotic progression [24, 25].

Pathophysiology of adhesive capsulitis remains incompletely understood. Pathological changes Have been observed in the joint capsule, coracohumeral ligaments, and synovial tissue. In addition to widespread fibrosis in the joint capsule, localized anterior capsule contracture Has also been observed in this syndrome. Previous investigations have revealed a decrease in type 1 collagen formation in the joint capsule and an increase in precursors such as α1(I) mRNA and other collagen types (V, VI, VII, XV, XVIII) [24, 26]. Pathological examinations suggest a pathophysiological relationship involving ASIC receptors within the capsule.

ASIC (Acid-Sensing Ion Channels) receptors are trimeric ion channels that detect extracellular pH reductions and permit the influx of ions into the cell. These channels play essential roles in the nervous system, including pain perception, synaptic transmission, learning, and memory, while in peripheral tissues, they are primarily associated with inflammation, ischemia, and mechanosensation. Each ASIC subtype exhibits distinct pH sensitivity and tissue distribution, making them potential therapeutic targets in various disorders. Furthermore, an increased expression of melatonin receptors has been associated with the upregulation of ASIC receptors [15]. Investigations on pathological specimens have revealed an increase in subtype ASIC3 channels in the capsule in patients with frozen shoulder syndrome. It is believed that these receptors play a role in pain formation and that the nocturnal pain observed in frozen shoulder syndrome is associated with melatonin [9, 27]. 

The effects of several well-known fibrotic agents, such as carbon tetrachloride, radiation, formalin, and cyclosporine, are attenuated by melatonin. Melatonin’s impact on the pathogenesis of fibrosis begins at the earliest stage [3, 28]. Furthermore, it has been demonstrated that melatonin reduces hepatic fibrosis by inhibiting necroptosis, highlighting its multifaceted role in mitigating fibrotic processes [18, 19].

In this subject, notable disparity in fibrosis detected within the shoulder joint capsule at the 16th week of the study suggests the efficacy of melatonin’s anti-fibrotic properties in adhesive capsulitis. This assertion is supported by the substantial contrast in fibrotic intensity between the groups: the first group which received the melatonin antagonist exhibited significantly higher fibrosis levels, whereas the group receiving melatonin showed lower levels. The absence of statistically significant differences in other parameters assessed in the histopathological examinations conducted during this period implies that melatonin primarly exerts its effects through the modulation of fibrosis. Subsequently, considering the 8-week period following the commencement of daily i.p. melatonin treatment for all groups and the histopathological examinations conducted afterward, the lack of statistically significant differences in any parameter among the groups indicates the therapeutic potential of melatonin. Even in the first group, which exhibited high-intensity fibrosis, no significant differences were observed in any parameter compared to the group that received treatment from the outset.

In evaluations conducted on the unaffected sides of all groups, the absence of a statistically significant difference in the total score and sub-parameters indicates that the effect of melatonin is weak in healthy shoulders and dominant in diseased areas. Hence, as demonstrated in previous studies, it is relatively safe for use in healthy individuals [29, 30]. Although a statistically significant difference was observed in the total score and fibrosis parameter score between the affected and healthy sides in Groups 1 and 3 at week 16, this difference became statistically insignificant at week 24 after treatment, clearly demonstrating the effectiveness of melatonin.

According to the results of this study, the use of melatonin in the frozen shoulder syndrome model created on experimental animals was successful. In adhesive capsulitis, which is common and can accompany other shoulder pathologies, and where conservative treatment is prioritized, we believe that melatonin can be used in all three phases due to its dominant anti-fibrotic effect and other beneficial properties. The fact that this study was conducted on rats, which are nocturnal (active at night) animals, may raise questions about the effect of melatonin on sleep in human studies.

The timing of melatonin administration—30 min prior to lights-off—was intentionally chosen to align with the natural circadian rhythm of rats, whose endogenous melatonin secretion typically begins shortly before the onset of darkness. This approach aimed to potentiate the hormone’s physiological effects by synchronizing exogenous supplementation with peak endogenous activity.

While this time point was selected based on prior circadian rhythm studies in rodents, future studies should consider evaluating the pharmacokinetics and tissue-level receptor activity at varying time points to fully elucidate the optimal therapeutic window for melatonin in fibrotic models.

In this study, the use of nocturnal animals such as rats—whose melatonin secretion naturally peaks during the dark phase—allowed for hormone administration to be timed in alignment with endogenous rhythms. Delivering melatonin prior to this peak may have enhanced its receptor affinity and downstream anti-fibrotic signaling [19, 31]. 

Melatonin was administered intraperitoneally to each animal at a daily calculated dose. However, further studies are needed to optimize the dose for therapeutic use in humans and to determine the most appropriate route and method of administration. The high degree of presystemic elimination complicates the direct oral use of melatonin in humans. Except for a few rare case reports in the literature involving extremely high doses, melatonin has not been demonstrated to cause harmful side effects, even at high dosesor chronic uses [29, 30, 32]. Future studies may consider exploring and optimizing the effects of intravenously or intra-articularly administered doses.

Several limitations are acknowledged in this study. Firstly, it was conducted as an animal experiment, and the induction of adhesive capsulitis was performed by the researchers. Additionally, the removal of scapulohumeral sutures through a second surgical procedure only 8 weeks after modeling may be considered a limitation.

The complex etiology and pathogenesis of adhesive capsulitis in humans are not fully understood, and the differences in disease development and healing between humans and the rat model remain unclear [24, 33, 34]. Furthermore, adhesive capsulitis in humans often coexists with other systemic and shoulder pathologies, which were not replicated in this model. The effects of melatonin on the sleep-wake cycle may differ between humans and nocturnal animals like rats. However, we do not consider this a limitation, as the focus of this study was to investigate the anti-fibrotic effect of melatonin rather than its influence on sleep regulation and circadian rhythm.

While improvements in certain histopathological indicators such as synovial thickening and total score reduction were observed in the control group, it is important to acknowledge the potential contribution of spontaneous recovery. Adhesive capsulitis in both human and animal models can exhibit a degree of natural resolution over time.

However, when comparing the magnitude and speed of improvement between the control and melatonin-treated groups, the observed histopathological changes—particularly the significant reduction in fibrosis scores—were more pronounced in the melatonin group. This suggests that while natural recovery may have contributed partially, the anti-fibrotic effects of melatonin likely played a dominant role. Future studies may benefit from including a sham-operated group or delayed-treatment group to more precisely distinguish between spontaneous healing processes and therapeutic intervention effects.

In response to the observed discrepancy in the number of euthanized animals at weeks 16 and 24, we acknowledge that three animals (two from the melatonin antagonist group and one from the control group) did not survive until the second endpoint. These losses were primarily attributed to postoperative complications and unrelated pathological conditions, as detailed in the Results section. While the study initially planned for equal subgroup distribution (*n* = 30), the mortality rate was 10%, which aligns with acceptable limits for surgical animal models involving shoulder immobilization.

To minimize potential selection bias, euthanasia at week 16 was performed randomly among the animals surviving the first 16 weeks, and the subsequent allocation for continued treatment was based on the remaining individuals. Therefore, the reduction in numbers at week 24 was not due to selective sampling but to natural attrition within the experimental limits.

Furthermore, the power analysis conducted prior to the study justified the sample size per group, ensuring statistical validity despite minor loss. Nevertheless, the limitation posed by reduced group size is acknowledged, and future studies with larger cohorts and loss-compensation strategies (e.g., oversampling) are warranted to reinforce these findings.

Another limitation of this study is the absence of additional quantitative techniques such as ELISA, Western blotting, or immunohistochemistry. Instead, histopathological evaluation was performed using hematoxylin–eosin and Masson’s trichrome staining, both of which are well-established and widely utilized in experimental pathology. Importantly, all specimens were blindly assessed by two independent pathologists, ensuring objectivity and consistency. While hematoxylin–eosin staining offers critical insights into general tissue architecture and remains fundamental for standardized histological interpretation, Masson’s trichrome provides robust information regarding extracellular matrix remodeling, making it particularly suitable for evaluating tissue-level fibrotic responses in experimental models.

Moreover, while adhesive capsulitis is a long-term disease with various stages, all rats in this study underwent surgery simultaneously, resulting in a uniform disease progression. The use of luzindole as a melatonin receptor antagonist in rats was chosen over pinealectomy to avoid additional morbidity and mortality, however luzindole may not neutralize all effects of melatonin. While the findings are promising, further randomized controlled trials are necessary to validate these results and determine the optimal dosage, administration route, and long-term effects of melatonin in human subjects.

## Conclusions

In conclusion, the study highlights the potential therapeutic role of melatonin in adhesive capsulitis, particularly in mitigating fibrosis progression and reducing synovial hypertrophy.

### Data availability

Datasets used and/or analysed during the current study are available from the corresponding author as a supplementary file.

## Supplementary Information

Supplementary Material 1

Supplementary Material 2

Supplementary Material 3

Supplementary Material 4

Supplementary Material 5

Supplementary Material 6

## Abbreviations

ARRIVE: Animal Research; Reporting of In Vivo Experiments
DMSO: Dimethyl Sulfoxide
i.p.: Intraperitoneally
